# Estimating the burden of rabies in Ethiopia by tracing dog bite victims

**DOI:** 10.1371/journal.pone.0192313

**Published:** 2018-02-21

**Authors:** Tariku Jibat Beyene, Monique C. M. Mourits, Abraham Haile Kidane, Henk Hogeveen

**Affiliations:** 1 Business Economics Group, Wageningen University, Wageningen, The Netherlands; 2 College of Veterinary Medicine and Agriculture, Addis Ababa University, Debre Zeit, Ethiopia; 3 Center for Outcomes Research and Epidemiology, College of Veterinary Medicine, Kansas State University, Manhattan, KS, United States of America; 4 Ethiopian Public Health Institute (EPHI), Zoonosis research unit, Addis Ababa, Ethiopia; 5 Department Farm Animal Health, Faculty of Veterinary Medicine, Utrecht University, Utrecht, The Netherlands; Scientific Institute of Public Health (WIV-ISP), BELGIUM

## Abstract

In developing countries where financial resources are limited and numerous interests compete, there is a need for quantitative data on the public health burden and costs of diseases to support intervention prioritization. This study aimed at estimating the health burden and post-exposure treatment (PET) costs of canine rabies in Ethiopia by an investigation of exposed human cases. Data on registered animal bite victims during the period of one year were collected from health centers in three districts, i.e. Bishoftu, Lemuna-bilbilo and Yabelo, to account for variation in urban highland and lowland areas. This data collection was followed by an extensive case search for unregistered victims in the same districts as the registered cases. Victims were visited and questioned on their use of PET, incurred treatment costs and the behavioral manifestations of the animal that had bitten them. Based on the collected data PET costs were evaluated by financial accounting and the health burden was estimated in Disability-Adjusted Life Years (DALYs). In total 655 animal bite cases were traced of which 96.5% was caused by dog bites. 73.6% of the biting dogs were suspected to be potentially rabid dog. Annual suspected rabid dog exposures were estimated per evaluated urban, rural highland and rural lowland district at, respectively, 135, 101 and 86 bites, which led, respectively, to about 1, 4 and 3 deaths per 100,000 population. In the same district order average costs per completed PET equaled to 23, 31 and 40 USD, which was significantly higher in rural districts. Extrapolation of the district results to the national level indicated an annual estimate of approximately 3,000 human deaths resulting in about 194,000 DALYs per year and 97,000 exposed persons requiring on average 2 million USD treatment costs per year countrywide. These estimations of the burden of rabies to the Ethiopian society provide decision makers insights into the potential benefits of implementing effective interventions.

## Introduction

Rabies is a viral infection that infects all mammals. Estimations on the global burden of rabies indicate a health impact of 59,000 human deaths per year, a loss of 3.7 million DALYs per year and about 8.6 billion USD of economic losses mainly due to premature deaths (productivity losses) and post-exposure treatment (PET) costs [1].

Most of the human death cases occur in Asian and African countries [2]. Of these countries, Ethiopia is one of the worst affected [1], with domestic dogs being the major sources of the infection to humans [3]. Dog management is, however, poor and anti-rabies dog vaccination is generally limited to a small number of dogs found in urban settings [4]. The large dog population size in combination with poor dog management contributes to a high endemicity of canine rabies in Ethiopia [5].

Basic information on the health and economic burden, e.g., on the amount of lives lost due to rabies and on the costs of treatment amongst those at risk, is crucial in the development of sustainable control programs [6]. In most rabies endemic countries, reliable reports of incidence data on rabies and rabies exposure are lacking. Official reports generally underestimate the true number of human rabies cases and hence the true burden [1; 7]. For instance, in sub-Sahara African countries like Tanzania, the incidence of human rabies predicted on the basis of active surveillance data on bite incidences was up to 100 times larger than the officially recorded number of deaths [8]. In Ethiopia, the national annual estimates from official reports indicate 12 exposure cases per 100,000 population and 1.6 rabies deaths per 100,000 population [9]. However, the actual numbers are expected to be higher as many cases are not reported [10].

The reliability of the reported incidence data is expected to differ among the regions in Ethiopia due to geographical as well as cultural differences. For instance, in rural Ethiopia, individuals who are exposed to rabies often prefer to see traditional healers for the diagnosis and treatment of the disease because of cultural background, lack of knowledge or limited accessibility to medical treatment. These widespread traditional practices of handling rabies cases might interfere with medical treatment seeking practice, resulting in an underreporting of the actual number of rabies cases and its related health burden. To fill the disparities between officially recorded and likely occurring rabies cases, researchers have applied approaches like extensive animal bite case searching, and predictive modeling [11;12;13; 14].

Rabies in exposed humans is preventable when an effective post-exposure treatment (PET) is applied immediately after exposure which includes post- exposure prophylaxis (PEP), wound washing, antibiotic and tetanus antitoxin administration. With respect to the applied PEP vaccine WHO recommends to use inactivated rabies vaccines produced in cell culture or embryonated eggs (CCEEV) [15]. In Ethiopia, the PEP vaccine used to treat most of the rabies exposures is produced from nervous tissue composed of rabies virus-infected sheep or goat brain inactivated with phenol and also called Nervous Tissue Vaccine (NTV) [6; 16; 17]. This type of vaccine is less immunogenic than the CCEEV and known for its fatality rate and disability rate due to the occurrence of severe and sometimes fatal allergic encephalomyelitic reactions [2; 6]. The health burden of rabies in Ethiopia, therefore, not only includes years of life lost due to the premature death following upon infection but also years of life lost due to the premature death following vaccination by NTV and years of life lived with a disability as a consequence of neurological complications, following NTV administration [2].

At present, although rabies control associated working groups have been established across the regions of Ethiopia, there is no national strategic plan to clearly define targets for rabies control [18]. The relative burden of rabies compared to other neglected tropical diseases is also not known [19]. In countries like Ethiopia, where financial resources are limited, there is a need for quantitative data on the costs of certain diseases to support intervention prioritization [20; 21]. Moreover, quantification of the health burden enhances the understanding of its long term effects and of the comparative advantages of different levels of treatment and prevention. Therefore, in this study, we aim to estimate the status of rabies in Ethiopia by assessing its health burden and associated PET costs using data obtained by an extensive bite case search.

## Materials and methods

### Study design

A retrospective study was conducted to assess the burden of rabies by collecting data on the incidence of human rabies exposure over the period of one year (September 2013 to August 2014) through an extensive animal bite case search. Animal bite victims were traced using data collected from the recorded cases at health centers as well as by information obtained from questioning the local community to trace unregistered bite cases. After the tracing, victims (or their family) were contacted and questioned about their use of PET, the health impact of the bite, the incurred costs and on the behavioral manifestation of the biting animal. Based on these data, the public health burden of rabies as well as the costs incurred because of the applied PET were estimated.

### Description of the study area

**Fig 1:**
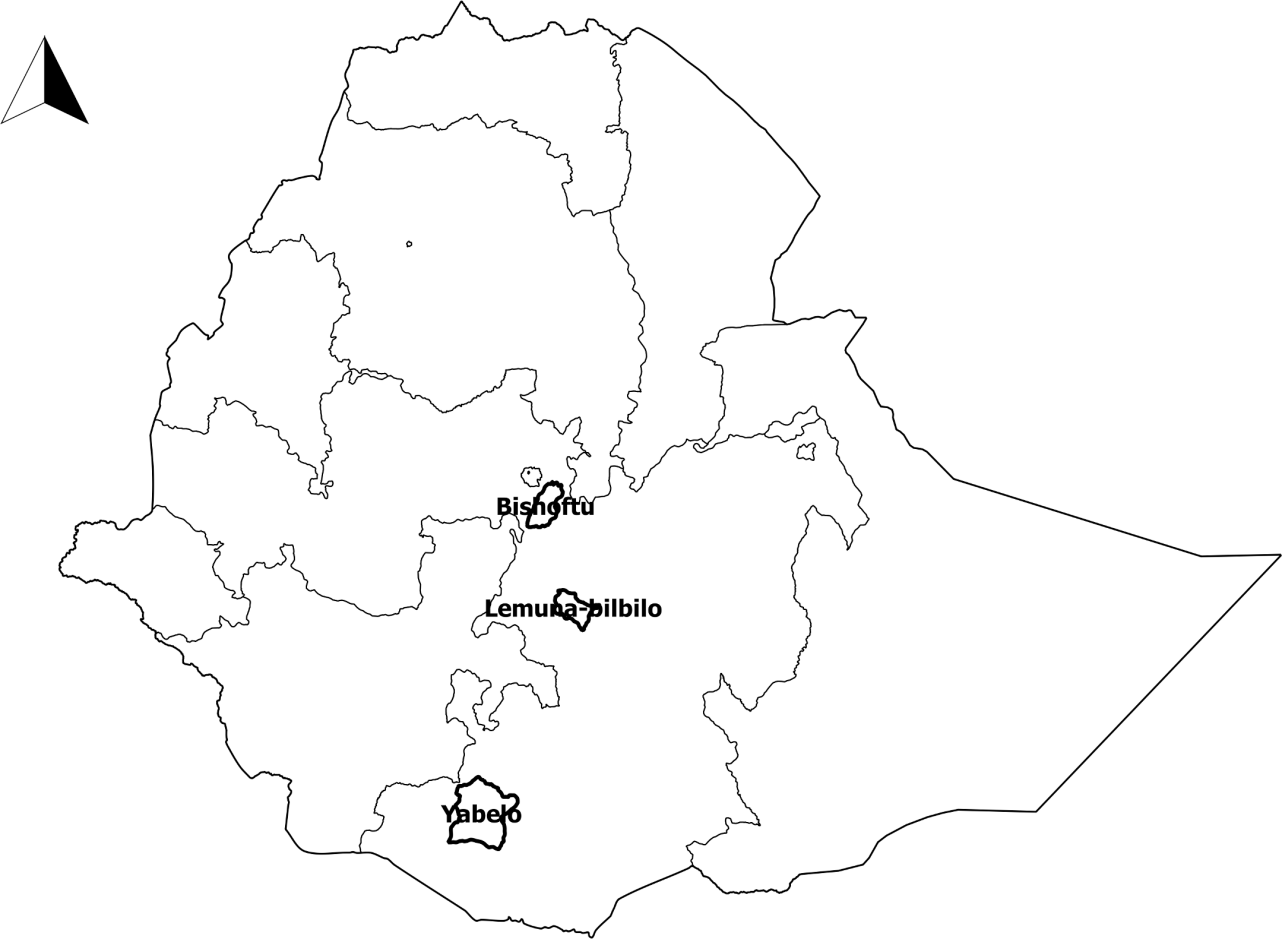
Map of Ethiopia showing the three study districts.

This study was conducted in three purposively preselected districts of Ethiopia, i.e. Bishoftu, Lemuna-bilbilo and Yabelo to account for the geographical variation within the country (Fig 1). Bishoftu is an urban district located at 45 km South East of Addis Ababa, the capital, with a relatively good infrastructure and health centers available within a reasonably short distance. Lemuna-bilbilo is a rural district located in the central highlands of Ethiopia, where most people live on a mixed crop and livestock farming system. Yabelo is a rural district in the lowlands of southern Ethiopia, where the majority of the people are pastoralists keeping livestock for their living. Both rural districts have poor road and health facility coverage. Bishoftu, Lemuna-bilbilo and Yabelo contain, respectively, 140000, 187000 and 101000 inhabitants [22], located in, respectively, 9, 27 and 25 villages/administrative divisions. Each district has one or more public health centers delivering medical services including PET and every village within a district has local health extension workers.

### Data collection

A list of animal bite victims registered by the districts’ health centers for the period of one year between September 2013 and August 2014 was obtained. Animal bite incidences were claimed in 55 out of the total of 61 villages in the three districts. In September 2014, local health extension workers of all villages, including those from the 6 villages without any registered suspected animal bite cases, were trained in techniques of detailed questionnaire interviews as described by Hampson et al., [14]. After this training, the extension workers contacted and interviewed all registered dog bite victims using a structured questionnaire. In cases where the victim was deceased or a child, an adult family member was interviewed. The health extension workers also performed an extensive search for non-registered animal bite victims by asking the contacted victims whether they knew other people that were bitten but did not visit a health center. In Ethiopia, most neighborhoods are socially close to each other and are therefore well informed about events happening to households in their village. Something serious as suspected rabid animal bite cases would, therefore, be known within the community [23]. The indicated non-registered bite victims were subsequently contacted and interviewed in the same manner. The questionnaire in English as well as translated to the local language (Oromifa) is attached as supplementary information (S1 File).

### Questionnaire survey

The bite victims were interviewed using a structured questionnaire on: 1) demographic features including age and gender, 2) body part bitten, 3) characteristics of the dog that had bitten them to reflect its behavioural manifestation and ownership (either own, neighbours’ or unknown), 4) whether the victim visited a health centre and received PET, 5) whether the person died (within a month after exposure) or survived and 6) the expenditures incurred related to treatment of the bite. The behavioral manifestation and clinical signs of the biting dogs as described by the bite victims were subsequently used to categorize the dog as potentially rabid or non-rabid based on the six-criteria method of Tepsumethanon et al. [24]. The same procedure was followed in interviewing the non-registered bite victims or their families. To check whether the bite actually occurred during the study period of September 2013 and August 2014, cross checking questions were asked by referring the incidence to specific verifiable (family) events.

### Statement of ethical clearance and informed consent

The study protocol and consent procedure were approved by the scientific and ethical review committee of the Ethiopian Public Health Institute (Reference No. EPHI-6-13-824) (Ethical clearance approval letter is attached as Supplementary information (S2 File)). An oral consent procedure was applied as illiteracy was expected among part of the respondent. Potential respondents (bite victims or their family members) were informed on the purposes of the research, the procedure followed during the interview and on the subsequent use of their responses. It was emphasized that their participation was completely voluntary and that they could stop with the interview at any time. All respondents approached agreed verbally to participate in the conducted interviews. Their consent was recorded (marked) on the questionnaire paper, which was used to register the answers of a respondent.

Permission to conduct interviews was also obtained from the district village health posts and administrative officials in all the study areas.

### Estimating the burden of rabies

The health and economic burden of rabies was estimated based on the cost classification of Jo [25] with the PET costs reflecting the direct costs, consisting of healthcare and non-healthcare costs expressed in monetary terms, and indirect costs in terms of DALYs as shown in Table 1.

**Table 1:** Cost classification for burden of rabies exposure.

Direct costs		Indirect costs
*Health care costs*	*Non-healthcare costs*	
• Diagnosis • Vaccine • Wound care and disinfection • Antibiotics • Tetanus immunization	• Transportation • Accommodation and food • Communication • Productivity loss /opportunity costs of time while seeking treatment	• Mortality (DALYs) • Morbidity (DALYs)

### Estimating post-exposure treatment costs

The costs of rabies post-exposure treatment were classified into two direct costs categories: 1) healthcare costs reflected by the expenditures for rabies vaccine, antibiotics, tetanus immunizations and disinfection (but not for rabies immunoglobulin as this was not used in the study areas) and 2) non-healthcare costs as the expenses for transport, food, and accommodation, while seeking PET (Table 1). Costs were estimated given the interview responses of the bite victims on the number of PEP doses received, the number of visits to the health centres to receive PEP (in case the patient or their parents was not sure or forgot the number of doses we asked the health centres) expenditures during the medical treatment related to transportation, accommodation, food and communication, and the time spent by themselves and their caregivers (if applicable) to account for productivity losses while seeking treatment.

For patients older than 15 years, the productivity losses while seeking treatment were valued in monetary terms using the GDP per capita daily income. When patients were accompanied during their treatment by an adult caregiver (older than 15 years), related productivity losses were also captured. Considering 568 USD as the Ethiopian average income per capita [26], we valued the daily average income at 1.6 USD per day. All the costs collected were in Ethiopian birr (ETB) and later converted to US dollars (USD) using the average annual exchange rate for the study period published by the National Bank of Ethiopia (1 USD = 19.22 ETB) [27].

For the estimation of PET costs, we considered PET costs per dog bite irrespective of the rabid status of biting dog and PET costs per sufficient treatment. The average PET costs per dog bite were defined as the sum of health care and non-health care costs including the opportunity cost of time spent while seeking for treatment divided by the total number of bite victims, including those who did not seek treatment.

A complete PEP dose of NTV consists of 17 doses of vaccine administered consecutively for the first 14 days, with the remaining 3 doses at intervals of 10 days i.e. at day 24, 34 and 44.

A treatment consisting of at least 14 of the 17 PEP doses was considered to be "sufficient", while any treatment consisting of less than 14 doses was regarded to be "insufficient", based on the minimum required number of doses to produce the neutralizing antibody level [28; 29]. Consequently, the average costs per sufficient treatment only accounted for the costs of patients who successfully received the minimum recommended doses (at least 14 out of the 17 doses) of PEP.

### Estimating the health burden of the disease

We adopted the disability-adjusted life years (DALYs) estimation method developed by the World Health Organization [30] to calculate the rabies health burden (Table 1). In accordance, DALY is the sum of years of life lost (YLL) due to rabies and adverse effects of NTV and of years lived with disability (YLD) due to adverse effects of NTV.

$$DALY=YLL+YLD=\overset{18}{\underset{i=1}{\sum }}\left(\left(Ndeath_{i}{*e}_{i}\right)+\left(Ndis_{i}*t*DW\right)\right)$$ (1)

YLL is calculated as the number of human deaths (*Ndeath*) within age category (*i*) multiplied by the life expectancy (*e*) of the concerned age category (*i*), cumulated over all age categories. Eighteen age groups with a 5 years age interval from 0 to 85 years and above were considered (*i* = 1…18) according to the age classification of WHO life expectancy table. The expected years of life lost at death were derived from the standard life table as given by WHO using the projected frontier life expectancy of 2050. Similarly, YLD is calculated by multiplying the total number of disability cases (*Ndis*) of the concerned age group (*i)*, with the duration of the disability (*t* in years) resulting from the NTV vaccine and its corresponding disability weight (*DW*) [2], cumulated over the 18 age categories. Computation was performed by the WHO programmed spreadsheet template for DALYs estimations [31; 32; 33; 34; 35; 36].

As the most recent global health estimates do not account for age weighting or time discounting, the baseline DALY estimation was done without these social weighting functions [31].

The YLL due to the disease were estimated from the number of deaths captured from the field survey. The disability (YLD) due to the disease itself was considered insignificant as the disease is very acute, leading to death once it develops [35]. The field survey primarily indicated the number of death due to rabies disease as specifically asked for. It was, however, not possible to derive a direct DALY estimation due to the adverse effect of NTV because the time lag between NTV administration and occurrence of adverse effects could have been longer than the duration of the field survey and because of the difficulty to attribute health problems specifically to the NTV application. As a consequence, the health burden due to NTV was indirectly derived from the number of persons receiving PEP corrected for the number of deaths due to rabies and estimated likelihoods of adverse effects based on the study of Knobel et al. [2]. YLL was derived from the number of patients who got at least one dose of NTV PEP, multiplied by the expected rate of neurological complications (0.4 out of 100 patients receiving NTV) and case fatality rate (17 out of 100 cases of neurological complications) [2]. The YLD due to NTV was calculated by multiplying the number of patients who got at least one dose of NTV PEP by the expected rate of neurological complications (0.4 out of 100 patients receiving NTV) and corresponding disability weight (DW = 0.613) for a most likely duration of disability of 8 days (t = 0.022 years) [2].

### Extrapolation districts estimates to national level

The health and economic burden of rabies was extrapolated to national scale by accounting for the proportion of the human population living in urban areas and rural areas. Of the total Ethiopian population of 95.7 million [22] only 19% lives in urban areas like Bishoftu. Information on the distribution of people among rural high and low land is lacking; hence the incidence estimates of both rural districts, Lemina-bilbilo and Yabelo, were used to obtain a general estimate for the rural area where 81% of the people are living. Under the assumption of a random occurence, stochastic estimates of the true incidence rates were estimated using the Poisson confidence interval. Given these confidence intervals, the sensitivity of the estimations on average health loss and treatment costs was evaluated.

### Statistical analysis

Descriptive statistics were used to summarize proportions of rabid suspected dog bite cases per age category, health center visits and sufficient doses of PEP receiving rates. Chi-square test and analysis of variance (ANOVA) were used to compare statistical difference between categorical variables and between group means. Tukey post hoc multiple testing for significance was used for additional exploration of the differences among means in cases of three or more group for which means were significantly different from each other. The statistical analysis was done in R statistical software (R 3.3.2) [37].

## Results

### Rabies exposure

A total of 655 bite victims was traced and interviewed with 210 cases in Bishoftu (urban), 262 cases in Lemuna-bilbilo (rural highland) and 183 cases in Yabelo (rural lowland) districts. From this total, 23 cases originated from bites by animals other than dogs, i.e. cat (2), cattle (7) donkey (7), horse (1), wild animal/rodents (3), and human (3). These cases were excluded from further analysis, as animal other than dogs contribute less than 4% of the total bites. Moreover, no proven means of diagnosing rabies in these animals without laboratory confirmation were available as for dogs [24]. Therefore, we focused on the remaining 96.5% cases originating from dog bites.

From the total number of dog bites, 208 (95.7%), 250 (61.6%) and 174 (56.3%) were reported to the health centers from, respectively, Bishoftu, Lemuna-bilbilo, and Yabelo,. The remaining cases did not seek medical attention and were identified through contact trace search. Based on the six- evaluation criterion of a biting dog [24], 189, 189 and 87 cases in the respective districts were found to be potentially caused by a rabid dog, indicating that on average 73.6% of the biting dogs were expected to be potentially rabid.

**Fig 2:**
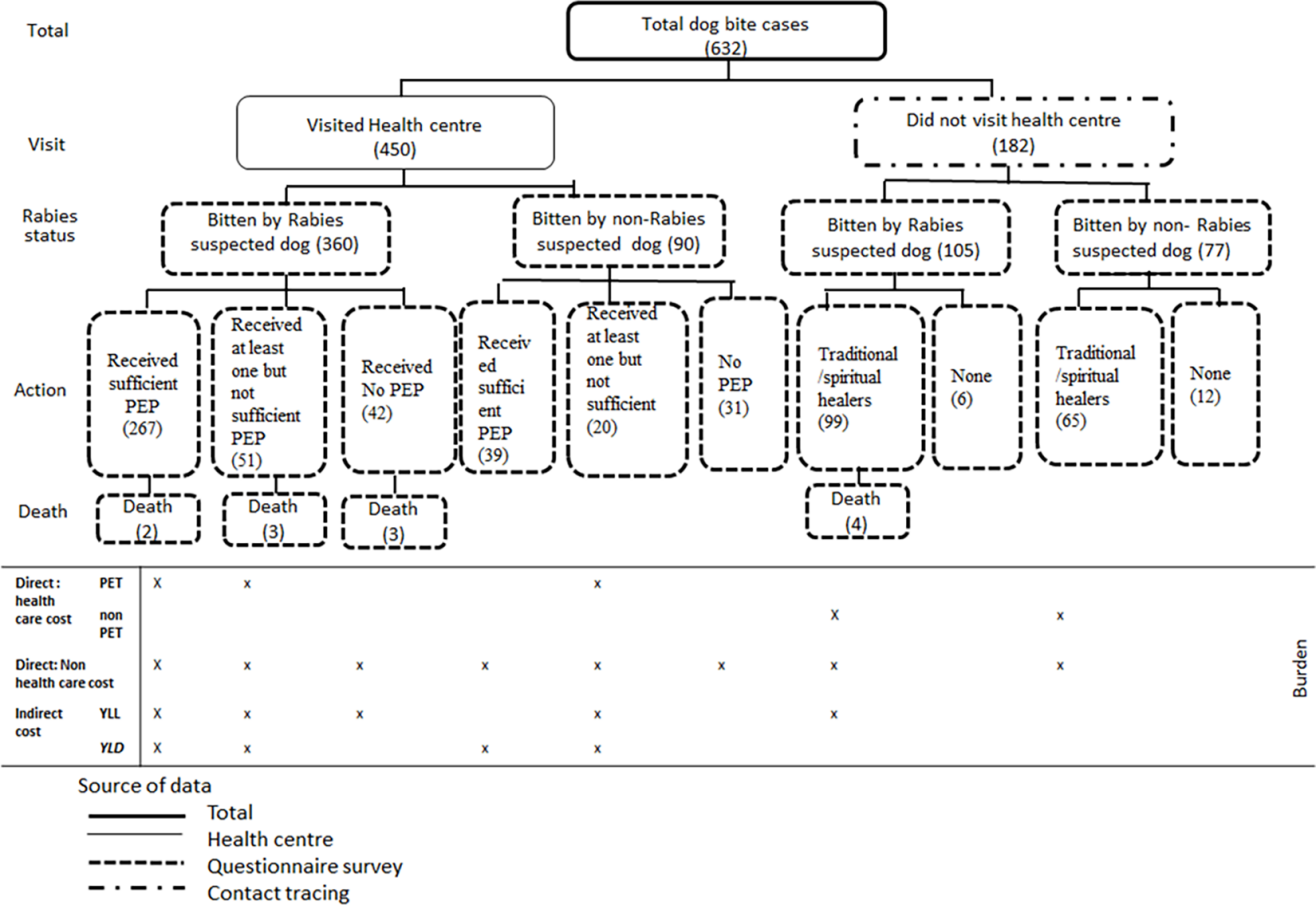
Classification of total dog bite cases to those who visited health center or not (visit), the rabies status of the biting dog and the type of treatment (action) with corresponding cost diagram underneath. Numbers of cases per category are indicated between brackets.

Fig 2 schematically presents the number of dog bite victims who visited a health center (450) and those that did not visit health center (182) as identified through the ‘‘snow balling” exercise during the contact tracing. All (632) bite victims were interviewed using a structured questionnaire. Out of these 632, according to our six evaluation criteria, 465 (73.6%) were bitten by a potentially suspected rabid dog of which 360 victims visited a health center and 105 did not visit a health center and consequently did not receive medical care. Victims who visited health centers received either a sufficient number of PEP doses or an insufficient number of PEP doses or no PEP at all. Similarly, victims who did not visit a health center either went to traditional healers or nowhere. All the 12 deaths resulted from bites from dogs suspected to be potentially rabid. The table below the schematic diagram presents the types of costs considered under each action taken (Direct health cost related to modern PET (post-exposure treatment) and non-PET (traditional/spiritual healers), Direct non-health care costs, and indirect costs in terms of DALY loss (Years of Life Lost (YLL) due to rabies and or adverse effect of the vaccine) and Years Lived with Disability (YLD) due to adverse effect of the vaccine).

When considering only rabid suspected dog bite cases, our data suggest that per 100,000 population, annually 135, 101 and 86 were exposed to potentially rabid dog cases in Bishoftu, Lemuna-bilbilo and Yabelo, respectively. Using chi-square test, we found a significant difference (p<0.01) in the incidence rate between the urban and rural districts. However, there was no significant difference between the highland (Lemuna-bilbilo) and lowland (Yabelo) rural districts (p>0.05).

The overall rabid suspected dog bite exposure ratio of male to female was 0.56:0.44. There were no statistical differences between the gender exposure rates in the urban area (Bishoftu), but significantly higher (p<0.01) exposure rates were observed in males than in females in both rural districts (p<0.01). The urban district (Bishoftu) has the highest rate of bite victims visiting a health center and receiving sufficient PEP compared to the rural districts (Lemuna-bilbilo and Yabelo). The proportion of rabies suspected dog bitten victims visiting health center was 97% in Bishoftu, 64% in Lemuna-bilbilo and 66% Yabelo. The proportion of suspected rabid dog bite victims receiving sufficient PEP was 78% in Bishoftu, 50% in Lemuna-bilbilo and 29% Yabelo. Conversely, in the same district order, 2.6%, 35.5% and 31% of the rabies suspected dog bite victims visited traditional/spiritual healers. Rabies-related human death cases compared to the number of total dog bite cases were higher in rural areas than in the urban area. Out of the 12 deaths counted during the survey, one was from Bishoftu, eight were from Lemuna-bilbilo and three were from Yabelo rural districts (Table 2).

**Table 2:** Number of dog bite cases, rabies suspected dog bite cases (RSDB cases), RSDB cases visiting a health center and RSDB cases receiving sufficient PEP (in absolute figures and relative to the number of RSDB cases (italic)).

	Total survey		Bishoftu		Lemuna-bilbilo		Yabelo	
Dog bite cases	632	*—*	208	*—*	250	*—*	174	*—*
RSDB cases	465		189		189		87	
RSDB visiting a health centre	360	*77*.*4*	183	*96*.*8*	120	*63*.*5*	57	*65*.*5*
RSDB receiving sufficient PEP	267	*57*.*4*	147	*77*.*8*	95	*50*.*3*	25	*28*.*7*
Rabies deaths	12	* *	1	* *	8	* *	3	* *

Age wise, the exposure to rabies suspected dogs in Bishoftu and Lemuna-bilbilo was highest in the 15–29 years age category and in Yabelo in the 5–14 years age category. Very young children and elderly people, i.e. 0–4 years of age and older than 60 years of age, were the least exposed age categories in all the districts. There was no significant difference among age groups across districts regarding the proportion of bite victims that received sufficient treatment (p>0.05). Overall, the age category of 15–29 was the group with the highest proportion of bite victims visiting a health center (85%) and receiving sufficient PEP (67%). In terms of visiting a health center and receiving sufficient PEP, the variations between age categories within district were significant in the rural districts (p<0.05) but not in the urban area (Table 3).

**Table 3:** Proportion of RSDB (rabies suspected dog bite) cases per age category.

		Districts		
	Total	Bishoftu/ Urban	Lemuna-bilbilo/ Rural Highland	Yabelo Rural-Lowland
Proportion of RSDB cases per age category				
0–4	3.7	3.7	4.8	1.1
5–14	30.5	27.5	29.1	40.2
15–29	38.5	44.4	35.4	32.2
30–44	16.8	14.3	19.0	17.2
45–59	6.9	6.3	7.4	6.9
>60+	3.7	3.7	4.2	2.3
Proportion of RSDB cases within an age category visiting a health center				
0–4	76.5	100	55.6	100
5–14	74.6	98.1	61.8	58.3
15–29	84.9	96.4	77.6	70.4
30–44	62.8	96.3	38.9	60.0
45–59	81.3	91.7	71.4	83.3
>60+	82.4	100	75.0	100
Proportion of RSDB cases within an age category receiving sufficient PEP				
0–4	64.7	71.4	55.6	100
5–14	52.8	80.8	45.5	22.8
15–29	66.5	79.8	61.2	39.3
30–44	44.9	77.8	33.3	13.3
45–59	59.4	58.3	64.3	50.0
>60+	47.1	71.4	37.5	0.0

An overview of bite cases and deaths by bite site and age of patient is given in Table 4. Out of the 465 rabies suspected dog bite cases, 6% occurred in the head/neck region, 19% on arms/hands, 70% on the legs and 6% on the trunk. Overall, proportions of RSDB cases were significantly different across body parts bitten with the highest proportion of bites on the legs and lowest proportion on the trunk. Based on age category, these proportions demonstrated a similar pattern except that for the age category beyond 15–29 years with the lowest proportions of bites on the head/neck part of the body. Of the 12 recorded deaths, 75% occurred in victims being bitten on the legs (Table 4). The two death cases following from a bite to the head/neck and the arms/hands received a sufficient number of PEP doses. Three of the deaths cases following from a dog bite on the trunk or the leg received an insufficient number of PEP doses, while the rest of the death cases did not receive any vaccine.

**Table 4:** Number of rabid suspected dog bite cases (RSDB cases) and deaths (indicated in brackets) according to bite site and age of the bite victim.

Age bite victim	Bite site				
	Head/neck	Arms/hands	Legs	Trunk	Total
0–4	3	4	9	1	**17**
5–14	17	23	94 (3)	9	**143 (3)**
15–29	2 (1)	36 (1)	132 (3)	8	**178 (5)**
30–44	2	12	59 (3)	5 (1)	**78 (4)**
45–59	1	6	21	4	**32**
60+	1	7	9	-	**17**
**Total**	**26 (1)**	**88 (1)**	**324 (9)**	**27 (1)**	**465 (12)**

### Rabies burden

#### Post-exposure treatment (PET) costs

The average PET costs per bite case were estimated at 21.3, 19.1 and 22.6 USD in Bishoftu, Lemuna-bilbilo and Yabelo, respectively. These estimates included health care and non-health care costs of all 632 dog bite cases which account for all type of treatment received (NTV, traditional or none) and did not significantly differ across the three districts (p = 0.12). The average total PET costs for those victims who received sufficient PEP doses of NTV were 23.4, 31.5 and 40.1 USD in the same district order. For these average costs per sufficient treatment, significant statistical differences were observed among the three districts.

Table 5 indicates the distribution of health care costs and non-health care costs per dog bite case and per sufficient treatment. In addition, S3 File indicates detailed description on the cost of treatment along with types of treatment. Generally, the non-health care costs account for the largest part in total PET costs. Healthcare costs per bite case were generally higher in the urban area than in the rural areas due to a higher application level of supplementary treatments such as tetanus antitoxins in addition to the basic wound care. The non-healthcare costs per sufficient treatment were higher in the rural districts than in the urban area as the distance traveled to the nearest health center was longer than in the urban area.

**Table 5:** Average (min, max) treatment costs (in USD) per dog bite (including all bite cases) and per sufficient PEP treatment.

	Average treatment costs per dog bite case (range)			Average costs per sufficient PEP treatment (range)		
	Bishoftu (n = 208)	Lemuna-bilbilo (n = 250)	Yabelo (n = 174)	Bishoftu (n = 149)	Lemuna-bilbilo (n = 113)	Yabelo (n = 44)
Healthcare costs	6.7 (0–30.3)	2.3 (0–18.9)	1.8 (0–31.3)	7.2 (2.3–30.3)	3.4 (2.3–18.9)	3.3 (2.3–14.8)
Non health care costs	14.6 (0–70.3)	16.8 (0–84.8)	20.7 (0–87.4)	16.2 (0–70.3)	28.1 (0–84.8)	36.8 (0–86.7)
**Total costs**	**21.3** **(2.3–72.7)**	**19.1** **(0–87.8)**	**22.6** **(0–91.62)**	**23.4** **(2.3–72.7)**	**31.5** **(2.3–87.8)**	**40.1** **(2.3–91.6)**

The largest portion of non-health care costs in the rural districts was attributed to the productivity losses due to the time spent on searching treatment. The number of days spent by victims in search of treatment ranged from 0 to 30 days per case with averages of 7.0, 9.0 and 8.4 days per bite case, and 8.5, 16.5 and 16.7 days per sufficient treatment in Bishoftu, Lemuna-bilbilo and Yabelo, respectively. Out of the total dog bite victims, about 35% was at least once accompanied by an adult family member (older than fifteen years of age) to the health center. The opportunity costs of time spent seeking for treatment per bite case ranged from zero to 54 USD with averages of 7.6, 10.0, and 8.7 USD for the victims and 1.6, 2.4, and 3.0 USD for the caregivers in Bishoftu, Lemuna-bilbilo and Yabelo, respectively. However, per sufficient treatment, the average opportunity costs of time seeking for treatment were equal to 9.2, 18.4, 18.1 USD for the victims and to 0.9, 3.5 and 4.1 USD for the caregivers in the same district order.

The number of days required to get a single PEP dose in the urban area was considered half a day, while in the rural areas it was about one day. The average number of days spent and the related opportunity costs while seeking treatment per case were comparable across districts due to a lower proportion of victims that was seeking for medical treatment in rural areas.

#### Health burden

Based on the total of 465 rabies suspected dog bites, the numbers of deaths due to rabies disease out of 100,000 population in Bishoftu, Lemuna-bilbilo and Yabelo districts were 0.7, 4.2 and 2.9, respectively. The health burden due to rabies was estimated to be 48.7, 297.5 and 239.8 DALYs per 100,000 population per year for Bishoftu, Lemuna-bilbilo and Yabelo districts, respectively. The numbers of human deaths and DALYs were significantly higher in rural areas compared to the urban area (p<0.05). Additionally, the estimated health burden due to the NTV application was 6.1, 2.9, and 2.9 DALYs per 100,000 population per year in Bishoftu, Lemuna-bilbilo, Yabelo, respectively. The largest contribution to this health burden resulted from YLL (i.e. 99%). The overall health burden (aggregated over the three districts) due to rabies and NTV was estimated to be 202.5 DALYs per 100,000 population per year (Table 6).

**Table 6:** Annual health burden due to rabies disease and adverse effects of Nervous Tissue Vaccine (NTV) in DALYs per 100,000 population.

Source of burden	Overall	Districts		
		Bishoftu	Lemuna-bilbilo	Yabelo
Rabies	198.6	42.6	294.6	236.9
NTV	3.9	6.1	2.9	2.9
Total DALY	202.5	48.7	297.5	239.8

#### National estimate

Given the surveyed death and exposure rates, the estimation on the true district incidence of rabies per year varied according to the Poission 95% CI as indicated in Table 7. Extrapolation of these estimations to the national level by weighing for the proportions of population living in urban (19%) and rural areas (81%), resulted in an estimated death rate of 3.0 cases per 100,000 at an estimated espossure rate of 101 cases per 100,000 (Table 7).

**Table 7:** Average district and national estimates on annual death rate and annual exposure rate per 100,000, indicated by average and possion 95% CI.

	Bishoftu	Lemuna-bilbilo	Yableo	National
Death rate	0.7 (0.0–4.7)	4.2 (1.1–10.2)	2.9 (0.4–8.0)	3.0 (0.6–8.8)
Exposure rate	135 (113–160)	101 (82–123)	86 (69–106)	101 (83–123)

With an Ethiopian population of 95.7 million these rates coincided with 2,871 (Poisson 95% CIs 574–8326) human rabies deaths and 96,657 (Poisson 95% CIs 78,474–117,711) rabies-exposed persons per year. When accounting for the uncertainty in incidence rates and considering average treatment costs of 21 USD per exposure case, the annual post exposure treatment costs at national level equalled an amount of 2 mln USD (1.6 mln– 2.5 mln). The corresponding national estimate on health loss due to the disease was estimated at 190,016 DALY per year (164,412–218,292). The health impact due to adverse effects of the NTV was estimated at 3,357 (765–9,091) DALY per year which made the total national health burden equal to 193,748 (168,049–222,406) DALY per year.

## Discussion

This study aimed to assess the burden of rabies in Ethiopia in terms of DALYs and PET costs while accounting for variation across different agro-ecologies. We recorded an average annual human mortality rate due to rabies between 0.7 (in Bishoftu) and 4.2 (in Lemuna-bilbilo) cases per 100,000 population with exposure rates ranging from 19–137 per 100,000 population with the highest number in Lemuna-bilbilo. Our evaluated numbers of exposures and deaths were higher than previous national estimates [9] consisting of 12 exposure cases per 100,000 population and 1.6 rabies deaths per 100,000 population, respectively. These estimates were calculated from confirmed exposure cases that were reported to health centers. Our estimates could have overestimated the actual number of bite cases by rabid dog as suspected cases were determined by the six-criterion method and not by laboratory confirmation techniques. The six criteria method has, however, been reported to be rather accurate (94.6%) [24] in the clinical diagnosis of rabies. Moreover, this retrospective study occurred up to a year after patients or family members had been bitten and were asked to provide details about the bite and the dog. Hence, responses could have been subjected to recall bias which would imply an under or over estimate of the burden. Recall error increases with the length of the recall period. Rabies is, however, one of the most feared diseases with almost 100% fatality rate. For this reason it was expected that the bite victims were rather accurate in remembering rabies exposures within the selected time frame of a calendar year.

Our suspected rabid dog exposure estimate was similar to the estimate made by Teklu et al., [38] in North-western Ethiopia but higher than the estimate of 1–5 exposure rates per 100,000 population as reported by Yibrah and Damtie [39]. Underreporting in Ethiopia primarily occurs due to the deep-rooted traditional practice of treating rabies by healers, which as such interferes with assessing the real magnitude of the problem [40]. In line with this, we found that about half of the bite victims we contacted in rural areas did not report to health centers but visited traditional or spiritual healers. In the urban area, this proportion was less than 4%. All exposure victims who seek treatment at least once but died later were not reported officially to the health centers or municipalities. This lack of follow-up by the health centers to monitor whether cases have recovered or died and been reported accordingly also contributes to the under reporting of rabies cases. The evaluated differences in treatment seeking behaviour among the urban and rural districts could, beside the differences in traditional practises, also be related to other socio-economic factors including difference in level of knowledge, accessibility to health centres or differences in the availability of financial means to call for further investigation (forthcoming paper Beyene et al.) in an attempt to improve the rate of sufficient PET in the rural areas.

The health burden of rabies ranged from 49–298 DALYs in urban and rural areas with an average of 3 human deaths per 100,000 population (Poisson 95% CIs 0.6–8.8), which is comparable to findings in countries in the region like Tanzania where 4.9 rabies caused mortalities led to 154 DALYs per 100,000 population [8]. Our study as well as the study in Tanzania [8], did not consider the temporary disability related to the time lived with severe open wounds which was given a disability weight of 0.005 (0.002–0.013) DALYs per patient [41]. The health burden due to the adverse effects of NTV is equivalent to 1% of the total health burden due to rabies disease. This estimate is comparable with a recent study on the global burden of rabies by Hampson et al., [1] reporting the neurological adverse effect constituting 0.8% of total rabies-related DALYs contributed by the 10 countries that reported to use NTV at the time of the study.

The health impact per 100,000 population and post-exposure treatment costs per sufficient treatment were estimated to be higher in rural districts. Consequently, the health and financial burden of rabies for rural victims were higher than the urban area, although the human rabies exposure rate to potential rabid dogs was higher in the urban district.

People in the age category between 15 and 29 years followed by children in the age between 5 and 14 years represented the largest groups among the rabid suspected dog bite cases. Children of 0–14 years of age had the highest probability of being bitten on the head/neck compared to other age groups. In most communities, children play with dogs. This predispose a higher risk for children to be bitten on the head/neck region. Exposure on upper parts of the body like head/neck is also associated with higher risk of developing the disease [2]. As acknowledged by WHO [30], the high life expectancy allocated to children and the elevated chance of exposure result in a higher burden of rabies in these age groups. Comparable proportions across age groups were reported by other studies for Northern Ethiopia [38] and countries in the East African region [14]. In the evaluated rural districts, males were shown to be at higher risk of exposure to rabies than females. This could be due to the socio-cultural influence of allocating most of the outdoor activities to males while females mostly work indoor. Similar findings were reported in other locations of Ethiopia [38; 39]

In this study, the average treatment costs per dog bite case irrespective of treatment status were more or less comparable among the districts. However, the proportion of health costs and non-health costs varied among the districts due to the fact that a higher proportion of the victims in the urban area sought medical treatment and needed to travel a shorter distance towards the nearest health center than victims in the rural areas. Health costs would generally increase if rabies immunoglobulin would also be administered for those who received PET. This is rarely applied in Ethiopian situation but recommended to all severe bite cases described by a single or multiple transdermal bites, scratches or contamination of mucous membrane with saliva (i.e. licks) [42]. The non-health care costs which include opportunity costs of productivity/time losses while seeking for treatment represented the most relevant costs for treatment. The non-health care costs varied across districts and were significantly higher in rural districts that in the urban districts. On average, the PET costs of rabies was equal to approximately 4% of the GDP per capita [43]. Considering only out of pocket spending, i.e. expenditures without considering the opportunity cost of time spent looking for treatment, the economic burden of PET would reduce with 50% to 2% of the GDP per capita, which is still a severe financial burden.

Application of the WHO recommended cell culture PEP by a 5-dose vaccine regimen [44] instead of the currently applied 17 doses NTV, would result in considerably higher health care costs (average of 62.5 USD per five doses WHO-PEP) versus 2.3 USD per 17 doses NTV-PEP). The non-healthcare costs will, however, be reduced due to the reduction in number of doses and therefore the required number of visits to the health centers (5 visits versus 17 visits). These costs would have been reduced further if the Essen 4 dose IM regimen was considered. An estimation of the non-health costs under the WHO regimen can be obtained from the average non-health care costs spent per PEP dose (i.e. treatment day) under the NTV regime. Accordingly, the average estimated PET costs of the WHO 5 dose regimen the average PET costs is estimated to be 71.8, 71.9 and 74.7 USD per sufficient treatment in Bishoftu, Lemuna-bilbilo, and Yabelo, respectively. This is approximately 3.5 times higher than the current average PET costs under NTV. This increase in costs is primary related to the difference in price of the imported cell culture based PEP and the locally produced NTV. Comparable costs were also reported in Tanzania for 5 doses of WHO recommended cell culture based PEP [45; 46]. Currently, the Ethiopian government is working towards establishing and producing cell culture based PEP locally that could lower the health care costs [47]. While the costs of cell culture vaccines are high, we only estimated a small saving of DALYs as a result of avoided adverse NTV effects; 6.1, 2.9 and 2.9 DALYs per 100,000 population per year in, respectively, Bishoftu, Lemuna-bilbilo, and Yabelo. Even though the health burden of the NTV compared to the total rabies burden is minimal, it could be eliminated by catalysing the current initiative of the Ethiopian government to replace the NTV to modern cell culture vaccine at the lowest cost possible.

Although the extrapolation to the national scale provides an indication of the health and economic burden in the country, the obtained estimate should be interpreted with caution, given the relative limited number of districts studied and the implicit assumptions made by weighting the district incidence rates to obtain estimates for the overall urban and rural areas. Our national estimate on the health burden is, however, in line with the estimation on the Ethiopian burden obtained from the most recent global rabies burden study [1] (175,945 DALYs).

In Ethiopia, priority has been given to diseases like Malaria, HIV/AIDS, and TB that attract funding for achieving Millennium Development Goals [35]. In addition, these diseases are rated as top priority in the current Ethiopian Growth and Transformation Plan (GTP). Although rabies is not comparable with these diseases which cause very high health and economic burden, our findings demonstrated that rabies causes a health and economic hardship and with a high likelihood of fatality for exposed rural inhabitants calling for national and global attention. Similarly, a recent study in Ethiopia [48] prioritized rabies as the most important zoonotic disease in Ethiopia. However, in practice rabies control is often overlooked by both public health and veterinary authorities. Considering the goal set by WHO to eliminate dog-mediated human rabies by 2030 [49] and meeting the Millennium Development Goals list of combating disease burden and reducing extreme poverty, including rabies control into the Ethiopian Growth and Transformation Plan would be very crucial.

Studies in sub-Saharan Africa countries, where rabies is a public health concern, have shown that investments made to vaccinate a sufficient proportion of dogs would likely minimize the health burden and expenditures related to PET [50; 51; 52]. Strategies to reduce human rabies in Ethiopia should, therefore, include mass dog vaccination. On the other hand, the implementation of a sufficient mass dog vaccination requires extensive knowledge of the dog population, the expected cost-effectiveness of vaccination measures and above all a strong integration of the public health and veterinary authorities for a progressive evaluation of one-health effectiveness in real time [53].

In conclusion, this study evaluated the health and economic burden of rabies in three districts representing geographical diversity assuming possible variation in human exposure rates. The health burden varied among the districts from 49–298 DALYs per 100,000 population with an average of 3 human deaths per 100,000 population (Poisson 95% CIs 0.6–8.8), Extrapolation of the results to the national level demonstrated that rabies, on average, causes 2 million USD treatment costs per year. These results indicate a relevant health and economic burden for Ethiopia. Human exposure rates to potential rabid dogs were higher in urban than in rural districts but the health impacts and post-exposure treatment costs were higher in rural districts. Consequently, the health and financial burden of rabies for rural victims are substantially higher than for urban victims. Under the current NTV regimen, non-health care costs are the major contributor of PET costs. Our estimations of the burden of rabies on the Ethiopian society could provide important information for evidence based decision making process with insights into the potential benefits of implementing cost-effective and coordinated intervention activities.

## Supporting information

S1 File Survey questions or questionnaire used in the study. (DOCX)

S2 File Ethical clearance approval. (PDF)

S3 File Detailed description on cost of treatment. (DOCX)
