# Supplementary material for: Estimating the burden of rabies in Ethiopia by tracing dog bite victims
Source: PLoS One. 2018 Feb 21;13(2):e0192313. doi: 10.1371/journal.pone.0192313 (PMC5821350; doi:10.1371/journal.pone.0192313)
Supplement: S1 File — (DOCX) [file pone.0192313.s001.docx]

**Gaafille qu’anoo/ Questionnaire to dog bite victims/their families**

Gaafii naamoota/maatii saree maraaten/tten ciniiname: Fulbaana 2013 - Hagaya 2014.

Questions to dog bite victims/their families exposed between September 2013 and August 2014

Maqaa nama gaafii gaafatu/*Name of enumerator* _________________________

Guyya itti gaafatame/ *Date of interview* ____________________________

Gafatamuf Fedhi qabaa/*respondent agreed to participate_*_____________________

**Kutaa I. Odeefanoo nama/maati ciniiname.**

- 1. Aanaa/*District*___________Ganda/*village*_____________
  2. Bara Dhalootaa _____________
  3. Umurii/*Age*:
  4. Saala/*Gender*: Dhiira Dhala*.*
  5. Amantii/*Religion*: Christian- Orthodox, Catholic, Protestant, Muslim, Other
  6. Sadarkaa barumsa/*Educational level of the victim:*

Ijoolee xiqqaa/*Underage child,* Hinbaratne/Illitrate, Elementary (0-6),

Sadarka lamaffa/*Juniour school* (7-10), sadarkaa olaana/*Highschool* (11-12), Kollejii/*College/university*.

- 1. Yeroo itti ciniiname/*Approximate day/month/year of during bite-*

1. **Seena beeladicha ciniine/***About the attacking/biting species*
   1. Goosa beeladichaa/*Biting species*:

A. Saree/*Dog* B. Adure/*Cat* C. Loon/*Cattle* D. Farad/*Horse*  E.Harre/*Donkey*

F. beeylada diidaa/*Wild animal* (__) G.Kaan/*Others*

- 1. Dhuma beeladicha/ *Subsequent fate of the attacking animal.*

A. Ajjefame/*killed,* B. bade/*disappeared*, C. Jirra/*alive*  D. Kaan/*other*

Yoo jirate/*If the available*- A. mirkana’e/ Confirmed B. hin mirkanoofne/*Not*, if confirmed- by sign or lab.

- 1. Abbumma beeladicah/ *Ownership of biting animals-*

1. Kessan/*Own,*  B. Olla/*Known neighbour,* C. lafa beekamu/*Known but far,*  D. hin beekammu/*unknown*

5. Mallatoole mula’atan/*Observed characteristics*

i) Umurii /*Age of the dog*?

a) Ji’a tokko gaddi/ *Less than 1 month* b) Ji’a tokko ol/*One month or more or not known*

ii) Haala fayyaa beeladicah?/*State of health of the dog?*

a) Fayya ykn guyya 10 ol dhukubsate/*Normal (not sick) or sick more than 10 days*

b) Guyya 10 gad dhukubsate/ Sick less than 10 days or not known

iii) Haala kaa’umsa dhukkubicha? *How did the illness evolve?*

a) Hattataman jalqabe/*Acute onset from normal health*

b) Suuta jalqabe/*Gradual onset or not known*

iv) Mallatoon isaa maal fakkata?/*How was the condition during the clinical course in last 3-5 days?*

a) Homaa hin fooyoofne/*Stable or improving (with no treatment)*

b) Fooya’a dhufe/*Symptoms and signs progressing or not known*

vi) Mallatoole kanaa gadii keessa yoo xiqaatte lama agarississa? *Does this dog show at least 2 of the 17 following signs or symptoms during the last week of life?*

a) Lakki/ *No, or showing only 1 sign*  b)Eyye/*Yes*

1. Mangaaga buussu/*Drooping jaw*

2. Sagalee adda/*Abnormal sound in barking.*

3. Arraba gogaa/*Dry drooping tongue.*

4. Finchaan ofiii araabuu/*Licking its own urine.*

5. Bishaan sodaachuu/*Abnormal licking of water.*

6. Alalfachu/*Regurgitation.*

7. Amala adda/*Altered behavior.*

8. Wantoota Ciniinuu fi Nyaachuu/*Biting and eating abnormal objects.*

9. Aaaruu/*Aggression.*

10. Otoo hin tuqamiin ciniinuu/ *Biting with no provocation*

11. Sababii malee cinimuu/ *Running without apparent reason.*

12. Yeroo fiigan goguu/ *Stiffness upon running or walking.*

13. Boqonna dhabuu/ *Restlessness.*

14. Of ciciniinuu/ *Bites during quarantine*

15. Kan rafe fakkachuu/ *Appearing sleepy.*

16. Hokoluu/ *Imbalance of gait.*

17. Hudduu dhaan taa’u/ *Frequent demonstration of the “Dog sitting”position.*

1. **Haala ciniinaa gadi fageegnaan.** *Characteristics of bite patients, details on site of bite and severity (WHO, 1988).*

a. Sadarkaa I – Tuqaa, Arrabuu (*touching or feeding animals, licks on the skin*).

b. Sadarkaa II - Madaa xiqoo (*nibbling of uncovered skin, minor scratches or abrasions without bleeding, licks on broken skin*).

c. Sadarkaa III – Madaa giddugaleesa fi gudaa (*single or multiple transdermal bites or scratches, contamination of mucous membrane with saliva from licks; exposure to bat bites or scratches*).

2. Qaama ciniiname/*On which body part are you bitten?*

a. Mataa ykn morma/ *Head/neck,*

b. Harka fi salgi/ *Arms,*

c. Qaama giddu/ *Upper trunk/lower trunck,*

d. Miila/ *Leg*

3. Qorsa aadaa argatee? *Did you get traditional treatment prior to health centre visit? Yes/No, If yes, what?*

4. Buufata fayyaa deemtee? *Did you visit to health centre?*

- Qorsa argatee? *Did you get treatment? Yes/no*

- Maaliif ? *If yes, what did you receive?*

1. Madaa dhiquu? *Wound washing*.
2. Hanga isaa hin beeku/ natti hin himamne. D*id not know the extent of treatment received.*
3. Kitibaata saree maraatee argadhera/ *Received rabies vaccine.if yes How many doses?*
4. Qorsa tetanesi argadhera/ *Received tetanus only.*
5. Qorsa tetanesi fi kitibaata saree matatee argadhera/*Received both rabies and a tetanus vaccine.*
6. Gorsa qofa/ *Only advice.*

5. Jia 1-2 keesa du’’e? Mo Jiraa? Died within 1-2 months or survived?

**Iv. Direct and indirect costs and coping strategies:**

- 1. Dhiimoota kanaa gadiif baasii hagam bastan?

Talaalli/*Vaccine:*

1. Gatii/*Price/dose.*

ii. Maddaa qulquleesu fi qorsa/*Wound care such as washing, antibiotics, tetanus immunizations and disinfection.*

3.3. Yoo tokko tokkon hin beekiin, Walumaa galatti hagam?

3. Dhiimoota kanaa gadiif baasii hagam baste?

i. Geejjibaaf/*Transport costs to and from health centres and hospitals,*

ii. Siiree fi bultii/*Accommodation.*

iii. Walquunamtii/*Communication (phone calls).*

iv. Nyaataafi dhugaatii/*Food and drinks*

v. Kaan/others.

4. Baasii ban biraa

i. Guyya haggam qorsa barbaadaa turte? *How many days you (the patient) spent in search of treatment?*
