# Supplementary material for: Estimating the burden of rabies in Ethiopia by tracing dog bite victims
Source: PLoS One. 2018 Feb 21;13(2):e0192313. doi: 10.1371/journal.pone.0192313 (PMC5821350; doi:10.1371/journal.pone.0192313)
Supplement: S2 File — (PDF) [file pone.0192313.s002.pdf]

የኢትዮጵያ የሕብረተሰብ ጤና ኢንስቲትዩት
Ethiopian Public Health Institute
አዲስ አበባ-ኢትዮጵያ Addis Ababa, Ehiopia

ስልክ-Tel: +251 11 2133499, +251 11 2751522, ፋክስ Fax: +251 11 2758634,
የመ.ሳ.ቁ - P. O. BOX: 1242/5654 e-mail: ephi@ethionet.et
www.ephi.gov.et

ቁጥር EPHI 6·13/824
Ref. No
ቀን 24 AUG 2015
Date

Mr. Tariku Jibat Beyene
Addis Ababa, Ethiopia

Subject:-**Approval of project proposal**

I would like to congratulate you and group that your Research proposal entitled **«Study on the burden of human rabies »**has been examined and approved for its scientific and ethical merits by our scientific and Ethical Review Committee.

Looking forward to seeing the best outcome of this work as a contribution to solving the health problem of our country, I wish you a successful implementation.

Sincerely yours ,

Yibeltal Assefa (MD,MSc,PHD)
Deputy Director General

Federal Democratic Republic of Ethiopia
Ethiopian Public Health Institute

CC:-
General Directer Office
SERO
EPHI

SCIENTIFIC AND ETHICAL REVIEW COMMITTEE- (SERC)

PROJECT REVIEW DECISION FORM

PROJECT TITLE: Study on the cost of treatment and public burden of rabies

PRINCIPAL INVESTIGATOR: Tariku Jibat

PROJECT NUMBER: SERO-026-8-2015; Version 001

APPROVAL DATE: August 7, 2015

APPROVAL DURATION: From August 7, 2015 to August 6, 2016

COMMENTS OF SERC

The project entitled "**Study on the cost of treatment and public burden of rabies**" has been reviewed for its benefit to the country, scientific validity and ethical standards. The objective of the project is to assess the incidence, costs and burden of rabies in Ethiopia.

The study is expected to generate evidence based information on socioeconomic burden of rabies in Ethiopia using one-health framework directing towards development of cost-effective and acceptable control strategy and eventual elimination of rabies from the country. The project proposal is found to be country relevant and scientifically validated. However, it requires data sharing agreement between EPHI and the institution to which the principal investigator is accountable in order to obtain the ethical clearance.

APPROVED [ ] CONDITIONALLY APPROVED [X] NOT APPROVED [ ]

SIGNATURE OF THE SERC MEMBERS

NAME	SIGNATURE
1. Getachew Addis	[signature]
2.	
3. Ibrahim Kedir	[signature]
4. Asfaw Debella	[signature]
5. Feven Girmachew	[signature]
6. Melke Tadesse	[signature]
7. Mekonnen Tadesse	[signature]
8.	

9. ______________________________________ ____________________

COMMENT AND FINAL DECISION OF THE INSTITUTE'S DIRECTOR

____

____

____

APPROVED [] *CONDITIONALLY APPROVED [X] NOT APPROVED [ ]*

*SIGNATURE*________________________*DATE* August 21/2015

Yibeltal Assefa (MD,MSc,PHD)
Deputy Director General
