# Supplementary material for: Estimating the burden of rabies in Ethiopia by tracing dog bite victims
Source: PLoS One. 2018 Feb 21;13(2):e0192313. doi: 10.1371/journal.pone.0192313 (PMC5821350; doi:10.1371/journal.pone.0192313)
Supplement: S3 File — (DOCX) [file pone.0192313.s003.docx]

S3 File: Detailed description on cost of treatment

|  | Average treatment costs per dog bite case | | | Average costs per sufficient PEP treatment | | |
| --- | --- | --- | --- | --- | --- | --- |
| Types of costs (Average estimates in USD) | Bishoftu | Lemuna-bilbilo | Yabelo | Bishoftu | Lemuna-bilbilo | Yabelo |
| Direct medical cost | 6.71 | 2.28 | 1.86 | 7.17 | 3.46 | 3.32 |
| Indirect medical cost (Transportation) | 5.39 | 2.41 | 4.39 | 5.90 | 3.26 | 6.59 |
| Indirect medical cost (Accommodation and food) | 0.05 | 1.99 | 4.58 | 0.07 | 2.97 | 7.97 |
| Indirect medical cost (Productivity loss) | 16.20 | 19.23 | 23.71 | 17.16 | 31.55 | 40.85 |
| Total expense | 21.36 | 19.11 | 22.55 | 23.36 | 31.53 | 40.10 |
